# *Per1/Per2* knockout Affects Spleen Immune Function in Elderly Mice via Inducing Spleen Lymphocyte Ferroptosis

**DOI:** 10.3390/ijms232112962

**Published:** 2022-10-26

**Authors:** Ruyi He, Shijie Zhang, Jiale Yu, Xiaojie Yu, Jian Wang, Yi Qiu, Wenting Zhou, Fangyi Wang, Feng Ren, Zhiguo Liu

**Affiliations:** School of Life Science and Technology, Wuhan Polytechnic University, Wuhan 430024, China

**Keywords:** circadian clock, *Per1/Per2*, spleen lymphocytes, ferroptosis

## Abstract

Disturbances in circadian rhythms are known to affect immune functions. However, the long-term impact of abnormal circadian rhythms on the immune-related functions of the spleen are poorly understood. Hence, we aimed to investigate the immune-related functions of spleen in *Per1/Per2* double-knockout (DKO) and wild-type (WT) mice aged 4, 9, and 14 months. Compared to the WT mice, the DKO mice had smaller spleen white pulp (WP) and lymphocyte germinal area, as well as fewer immune cells with age—these differences were especially clear. The spleen lymphocyte mortality, malondialdehyde (MDA) levels, reactive oxygen species (ROS) levels, and ferritin-binding receptor (TFR1) levels were significantly higher in the 14-month-old DKO mice than in WT mice of the same age. Transcriptome analysis showed that most of the differentially expressed mRNAs were enriched in DNA damage repair-related pathways. In DKO mice, spleen cells showed up-regulation of pro-ferroptosis genes, such as *Cd36,* *Atm*, and *Acsl4*, and down-regulation of anti-ferroptosis genes, such as *GPX4*. We found that long-term abnormalities in the circadian rhythm can induce DNA damage and ferroptosis in mouse spleen.

## 1. Introduction

Circadian rhythms influence the patterns of mammalian behaviors and physiology. An elaborate set of molecular mechanisms are regulated by the period genes, *Per1* and *Per2*, and cryptochrome genes, *Cry1* and *Cry2*, all of which control the rhythm of the biological clock. The PER and CRY proteins form heterodimers that inhibit the transcription of their genes by blocking the BMAL1/CLOCK activity [1]. These core clock proteins also form a regulatory loop with RAR-related orphan receptors (RORs) and nuclear receptor subfamily 1 group D member 1 (NR1D1, also known as Rev-Erb alpha) proteins for the regulation of the biological clock [2,3].

Disruption of the circadian rhythm can cause cardiovascular disease [4], gastrointestinal diseases [5,6], reproductive abnormalities [7], brain disorders [8], and immune system damage [9,10]. Recent work has found that cerebrospinal fluid (CSF) drains into the lymphatic system [11]. Therefore, waste clearance from the CSF may also be influenced by the circadian clock. In addition, circadian disruption can also lead to the loss or inversion of daily patterns of M1 (pro-inflammatory) and M2 (anti-inflammatory) macrophage activity and cytokine levels in spleen and tumor tissues. In tumors, this may promote the remodeling of the tumor microenvironment (TME) and favor tumor cell proliferation [12]. Thus, circadian disruption can severely affect the body’s lymphatic and immune systems. The spleen is the largest secondary lymphoid organ in the body, and is responsible for a wide range of immunological functions in addition to its roles in hematopoiesis and red blood cell clearance [13]. However, the impact of long-term abnormalities in circadian rhythms on the function of the spleen is poorly understood, especially in old age. We aimed to investigate if chronic circadian rhythm abnormalities impair spleen function in old age. This research is important for understanding the health issues of night shift workers in an increasingly aging society.

This study used double-knockout (DKO) *Per1/Per2* mice aged 4 (young), 9 (middle-aged), and 14 (old) months to explore the effects of circadian rhythm abnormalities on the spleen. Physiological indicators and hematoxylin–eosin (HE) staining showed that the older mice had significant and obvious spleen damage. Flow cytometry showed that the numbers of blood and spleen lymphocytes in 14-month-old DKO mice were significantly lower than those in WT mice of corresponding ages. While the differences in the numbers of blood and spleen lymphocytes in 9-month-old and 4-month-old DKO mice were minor, the differences in these numbers between DKO and WT mice of corresponding ages were significant. Transcriptome analyses of spleen tissues of 14-month-old mice revealed that *Per1/Per2* DKO induced DNA damage repair in the spleen, followed by lipid peroxidation and ferroptosis through multiple pathways, including induced ROS, up-regulation of *ACSL4* expression, and PUFA-PL (polyunsaturated fatty acid-containing phospholipids) peroxidation, along with dampening of glutathione peroxidase 4 (GPX4) expression. Immunohistochemical staining indicated that lymphocyte mortality, ROS, and MDA were higher in DKO mice than in WT mice. The levels of ferritin-binding receptor (TFR1) increased with age in the DKO mice. Interestingly, these pathways are also involved in radiotherapy-induced ferroptosis [14], which suggests that long-term abnormalities in the circadian rhythm can negatively affect the spleen’s immune function in mice.

## 2. Results

### 2.1. Effects of Per1/Per2 Knockout on Spleen Weight and Pathological Structure

**Figure 1:**
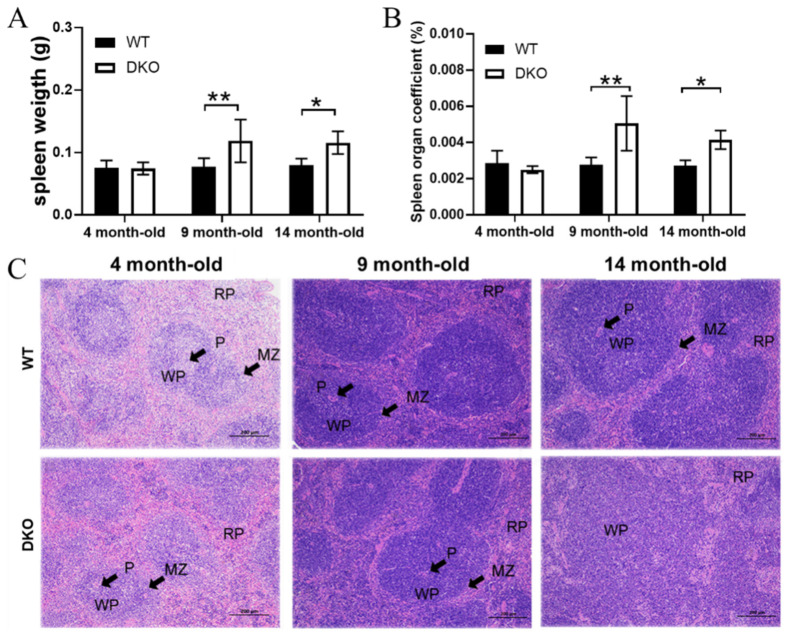
Physiological effect indices and HE staining of spleen from DKO and WT mice of different ages. (**A**) Spleen weight (g). (**B**) Spleen organ coefficient (%). (**C**) Spleen HE staining. White pulp (WP), red pulp (RP), periarterial lymphatic sheath (P), and marginal area (MZ), scale = 200 µm. Data are presented as mean ± SD, *n* = 20. * *p* < 0.05, ** *p* < 0.01.

In the 4-month-old mice, we observed no significant differences in either spleen weight or spleen weight–body weight ratios between the DKO and WT mice. However, in the 9- and 14-month-old mice, spleen weights and spleen weight–body weight ratios were significantly higher in the DKO mice than in WT mice (*p* < 0.05; Figure 1A,B).

The white pulp (WP) and the red pulp (RP) are two functionally and morphologically distinct regions of the spleen. The WP can be further differentiated into the periarterial lymphatic sheath (P) and the marginal zone (MZ), while the RP consists of the hyperemic sinuses and the splenic cord. In a normal spleen, there is a clear boundary between MZ and WP. However, pathological changes can lead to the blurring of this boundary. The spleens of DKO mice showed some pathological changes, which increased with age (Figure 1C); such changes were not as evident in WT mice. At 4 and 9 months, the DKO mice had a fairly normal spleen structure containing a loose WP structure with light staining and a clear boundary between the WP and MZ. However, by 14 months, the WP structure was loose and incomplete, the P was blurred or had disappeared, and the MZ boundary could not be seen. The spleen WP and lymphocyte germinal area in the DKO mice decreased significantly with age and were also significantly smaller than those of WT mice of the same age.

### 2.2. Levels of B Cells, CD3+-CD4+T Cells, and CD3+-CD8+T Cells in Blood and Spleen of DKO and WT Mice at Different Ages

**Figure 2:**
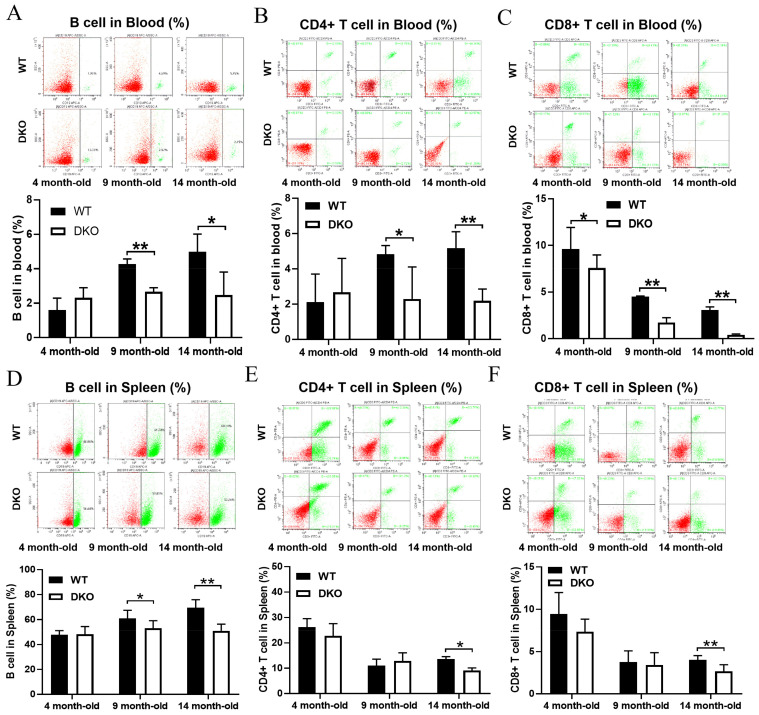
B, CD3+-CD4+T, and CD3+-CD8+T cells in the blood (**A**–**C**) and spleen (**D**–**F**) of DKO and WT mice at different months of age. Data are presented as mean ± SD, *n* = 6. * *p* < 0.05, ** *p* < 0.01.

Flow cytometry was used to measure the numbers of B cells, CD3+-CD4+T cells, and CD3+-CD8+T cells in the blood and spleen of WT and DKO mice aged 4, 9, and 14 months (Figure 2). All three cell types in blood were significantly lower in the 9- and 14-month-old DKO mice compared to WT mice of corresponding ages (Figure 2A–C). There were no significant differences in the number of B cells and CD3+-CD4+T cells in blood between 4-month-old DKO and WT mice (Figure 2A,B); however, the number of CD3+-CD8+T cells in blood was significantly lower in DKO mice than in WT mice (Figure 2C). In the spleen, there were no significant differences in the number of B cells, CD3+-CD4+T cells, and CD3+-CD8+T cells between 4-month-old DKO and WT mice (Figure 2D–F). B cell numbers were significantly lower in 9-month-old DKO mice than in WT mice (Figure 2D), but there were no significant differences in CD3+-CD4+T and CD3+-CD8+T cell numbers between the DKO and WT mice (Figure 2E,F). The numbers of all three cell types in 14-month-old DKO mice were significantly lower than those in 14-month-old WT mice (Figure 2D–F).

### 2.3. DNA Damage Response and Oxidative Stress Resistance in DKO Mice Spleen Tissue Detected through Transcriptome Sequencing

The total RNA contents of spleen cells from 14-month-old DKO and WT mice were sequenced and analyzed. The differentially expressed genes (DEGs) in DKO and WT mice were identified using principal component analysis (PCA) (Figure S1A). In total, 3583 genes were up-regulated and 2290 were down-regulated in DKO mice. Genes exhibiting differential expression levels > 0.1 (fold change in expression or log2FoldChange) and *p*-value < 0.05 were selected for further analysis (Figure S1B), and the results are presented as a heatmap in Figure S1C.

**Figure 3:**
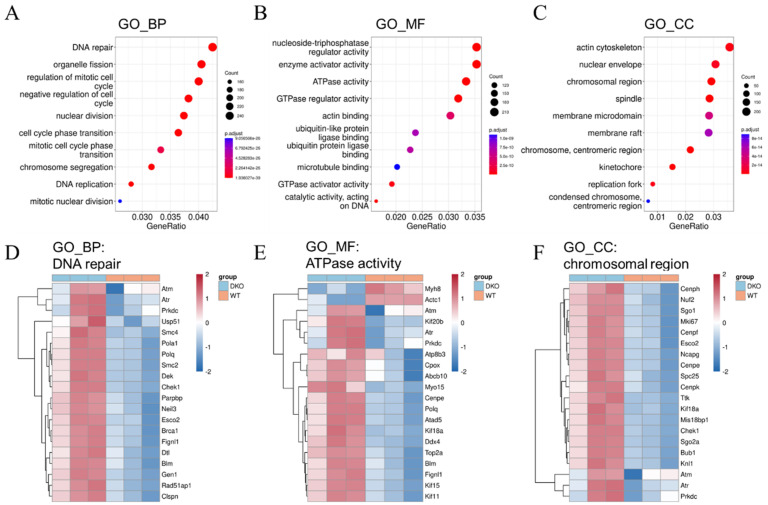
GO analysis of DEmRNAs in 14-month-old DKO male mice spleen. (**A**) GO-BP; (**B**) GO-MF; (**C**) GO-CC. Colored dots represent the fold change in gene expression in different pathways. The *y*-axis represents the gene count. The *x*-axis gives enrichment analysis terms. Plot colors represent *p*-values and sizes represent gene numbers. (**D**–**F**) Heatmap for differentially expressed mRNAs enriched in DNA repair (BP), ATPase activity (MF), and chromosomal region (CC). Blue: DKO mice; red: WT mice. DEmRNAs: differentially expressed mRNAs; GO: Gene Ontology; BP: biological process; CC: cell component.

Gene Ontology (GO) functional enrichment analysis was performed to determine the functions of the DEGs. The results showed that most of differentially expressed mRNAs (DEmRNAs) belonged to genes involved in the DNA damage response (DDR) pathway, including DNA repair (biological process, BP) (Figure 3A,D), ATPase activity (molecular function, MF) (Figure 3B,E), and chromosomal region (cell component, CC) (Figure 3C,F). Three DNA damage response genes, *Atm*, *Atr*, and *Prkdc*, were significantly up-regulated in the spleens of DKO mice (Figure 3D,F). In addition, the genes *Msh2*, *Msh3*, *Msh5*, *Msh6*, *Pms2*, *Dnmt*, *Apex2*, and *H2afx*, all of which are involved in DNA repair pathways, were also up-regulated in the spleens of the DKO mice (Figure S2A).

**Figure 4:**
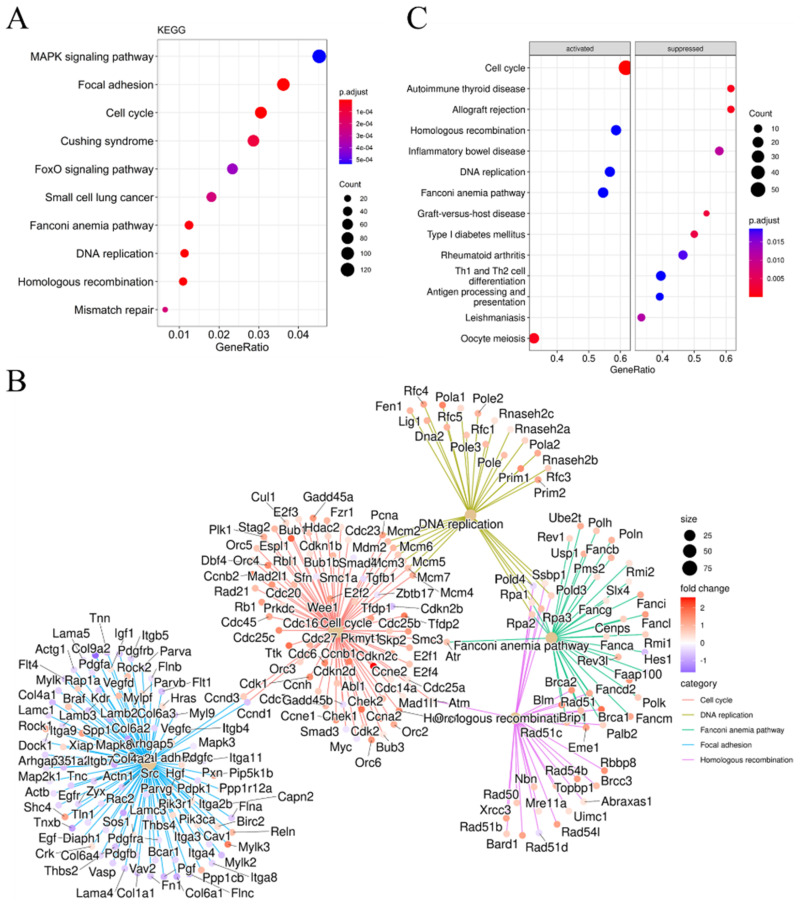
KEGG and GSEA analysis of DEmRNA in 14-month-old DKO mice spleen. (**A**) KEGG pathway analysis of mRNAs. Colored dots represent the fold change in gene expression in different pathways. The *x*-axis represents the gene ratio. The *y*-axis gives the enrichment analysis terms. (**B**) The net plot of KEGG pathways shows the enrichment of DEmRNAs in different pathways. Plot colors represent the *p*-values and sizes represent the gene numbers. (**C**) Gene set enrichment analysis (GSEA) for DEmRNAs. The *x*-axis represents the gene ratio. The *y*-axis gives the enrichment analysis terms. Plot colors represent the *p*-values and sizes represent the gene numbers. DEmRNAs: differentially expressed mRNAs; KEGG: Kyoto Encyclopedia of Genes and Genomes; GSEA: gene set enrichment analysis.

Kyoto Encyclopedia of Genes and Genomes (KEGG) analysis showed that some DEmRNAs belonging to genes involved in the FoxO (forkhead box O) signaling pathway (Figure 4A), such as *Fox3* and *Fox4*, were up-regulated in DKO mice. This pathway is involved in many cellular physiological processes, such as cell cycle control, immuno-regulation, and oxidative stress resistance. In contrast, genes belonging to the TGF-β signaling pathway, such as *Smad3*, *Smad4*, *Bcl6b*, *Sod3*, and *Gadd45b*, were down-regulated in DKO mice (Figure S2B); these genes are involved in immuno-regulation and oxidative stress resistance. A pathway–pathway interaction network (PPIN) analysis based on the enriched DEmRNAs in each pathway was constructed. Most DEmRNAs were enriched in five pathways, namely cell cycle, DNA replication, Fanconi anemia pathway, focal adhesion, and homologous recombination (Figure 4B), all of which are associated with DNA damage response. Gene set enrichment analysis (GSEA) (Figure 4C and Figure S3) also proved that cell cycle, DNA replication, and the Fanconi anemia pathway were activated in the spleens of DKO mice.

### 2.4. Differences in the Immune Microenvironment and Immune-Related Signatures between the DKO and WT Mice Spleen Based on Transcriptome Analysis

**Figure 5:**
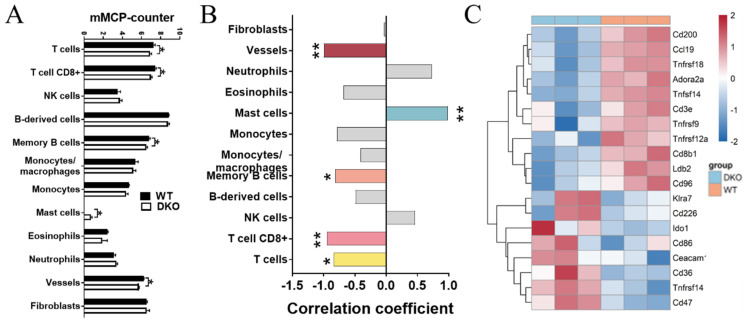
Differences in the immune microenvironment between the DKO and WT mice spleen. (**A**) The mMCP-counter of different immune and stromal cells in DKO and WT mice spleen. Data are presented as mean ± SD, *n* = 3. * *p* < 0.05. (**B**) The correlation between DKO score and immune cell infiltration in mice spleen. * *cor_pvalue* < 0.05, ** *cor_pvalue* < 0.01. (**C**) The differential expression of immune checkpoint genes in DKO and WT mice spleen. Blue: DKO mice; red: WT mice. mMCP-counter: murine Microenvironment Cell Population counter.

We then calculated and investigated the correlation between *Per1/Per2* score (DKO: 0; WT: 1) and immune cell infiltration based on 14-month-old mice spleen transcriptome data using the R package “mMCP-counter” (Murine Microenvironment Cell Population counter) [15]. We found that the DKO mice had lower numbers of immune cells (T cells, CD8 T cells, and memory B cells) and stromal cells (vessels) (Figure 5A,B), consistent with our flow cytometry results (Figure 2). We further examined the relationship between the Per1/Per2 score and the expression level of immune checkpoint genes. Notably, CD200 [16], a classic immune checkpoint that is expressed on the cell surfaces of B cells and activated T cells, was down-regulated in the DKO mice (Figure 5C). In contrast, CD36, which could mediate ferroptosis and dampen intratumoral CD8+ T cell effector functions to impair their antitumor ability, was up-regulated [17]. Thus, Per1/Per2 DKO could cause a massive loss of immune cells in the spleen of 14-month-old mice.

### 2.5. Per1/Per2 Knockout Activated Ferroptosis in the Spleen of 14-Month-Old Mice

**Figure 6:**
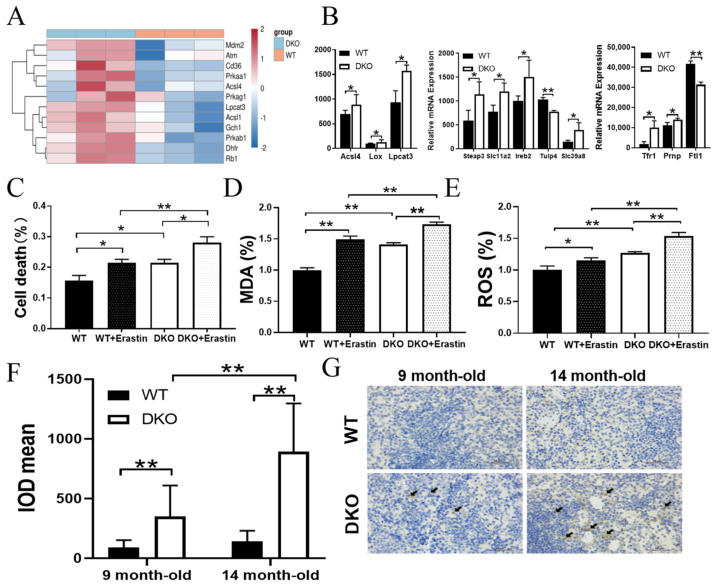
Expression analysis of spleen tissue and spleen lymphocytes. (**A**) Heat map of spleen DNA damage and ferroptosis gene expression levels in 14-month-old mice. (**B**) Relative mRNA expression of lipid peroxidation-related genes and iron metabolism-related genes. *n* = 3, * *p* < 0.05. (**C**) Cell mortality, (**D**) MDA levels, and (**E**) ROS levels of WT and DKO spleen lymphocytes from 14-month-old mice as well as treatment with 0.5 µM erastin. Data are presented as mean ± SD, *n* = 6. * *p* < 0.05, ** *p* < 0.01. (**F**,**G**) IHC staining of spleen TFR1 in mice aged 9 and 14 months. Data are presented as mean ± SD, *n* = 3. ** *p* < 0.01.

Analyzing the ferroptosis-inducing genes in the spleen cells of 14-month-old DKO and WT mice (Figure 6A), we observed that most ferroptosis activators, including Prkaa1, Prkab1, Prkag1, Cd36, Acsl1, Dhfr, Gch1, Rb1, Mdm2, and Atm, were up-regulated in the DKO mice. Next, we analyzed the lipid peroxidation-related genes and iron metabolism genes (Figure 6B). The results indicated that the expression levels of Acsl4, Lpcat3, lipoxygenase (Lox), Tfr1, Prnp, Steap3, slc11a2, Ireb2, and Slc39a8 [18] were higher in the DKO mice than in WT mice. In contrast, the iron storage genes Ftl1 and Tulp4 [19] were down-regulated in the DKO mice compared with the WT mice (*p* < 0.05, Figure 6B and Table S1).

Compared with WT, the splenic lymphocyte mortality, MDA levels, and ROS levels were increased significantly in DKO mice (*p* < 0.05, Figure 6C–E). In addition, these indicators in primary lymphocytes of DKO mice stimulated with erastin (one of the activators of ferroptosis) incubation were significantly higher than those of similarly stimulated primary lymphocytes of WT mice (*p* < 0.05; Figure 6C–E). These results indicated that the spleens of DKO mice were more susceptible to oxidative damage.

Immunohistochemical staining of TFR1 in spleen sections obtained from 9- and 14-month-old DKO and WT mice (Figure 6F,G) indicated that TFR1 expression was significantly higher in DKO than in WT spleen tissues. In addition, the spleen sections from 14-month-old mice had significantly higher TFR1 levels than those of 9-month-old mice.

### 2.6. Per1/Per2 DKO Promotes Ferroptosis in the Spleen of 14-Month-Old Mice

**Figure 7:**
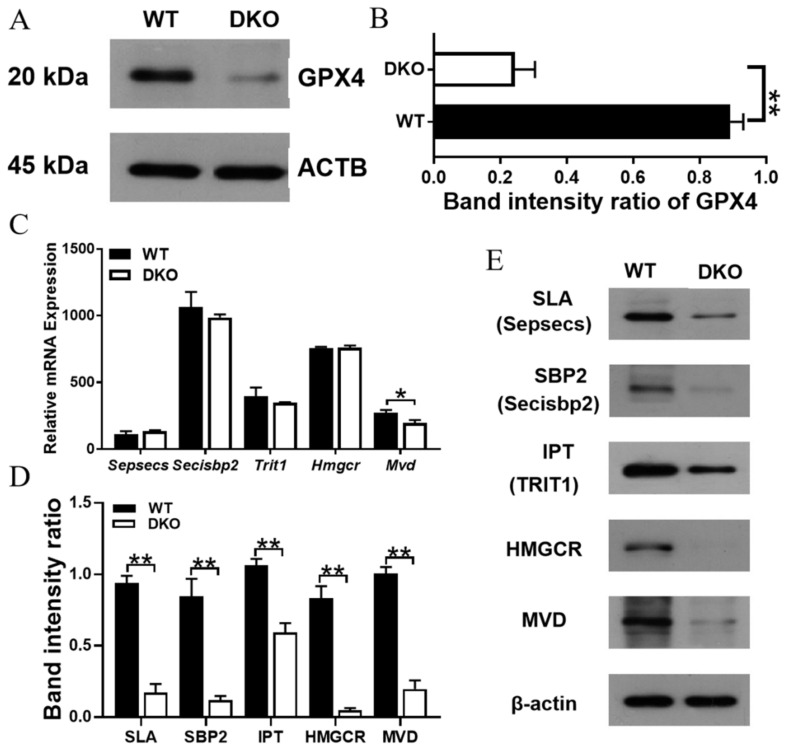
Analysis of GPX4 synthesis regulation in 14-month-old mice. (**A**,**B**) GPX4 protein expression in WT mice and DKO. Data are presented as mean ± SD, *n* = 2. ** *p* < 0.01. (**C**) Relative mRNA expression and protein expression (**D**,**E**) related to the pathway of mevalonate metabolism and selenoprotein synthesis in WT mice and DKO. Data are presented as mean ± SD, *n* = 3. * *p* < 0.05, ** *p* < 0.01.

Western blot analysis indicated that the levels of GPX4, a key regulator of ferroptosis, were significantly lower in the splenic lymphocytes of 14-month-old DKO mice than in those of WT mice (*p* < 0.01; Figure 7A,B). In addition, the transcriptome levels of Hmgcr, Mvd, Trit1 (IPT), Sepsecs (SLA), and Secisbp2 (SBP2) genes were not changed, except Mvd (Figure 7C, Table S1), but the levels of corresponding proteins were down-regulated in 14-month-old DKO mice spleens as compared to those in 14-month-old WT mice spleens (*p* < 0.05; Figure 7D,E). RT-PCR and RNA sequencing analyses indicated that Rorγ and Srebp2 were also down-regulated in DKO mice (Figure S4, Table S1).

## 3. Discussion

Previous studies have demonstrated the impact and regulatory influence of circadian rhythms on immune functions [20]. In addition, the age-related declines in cellular immune functions and humoral immune responses may partly explain the increased prevalence and severity of infectious diseases, and the impaired effectiveness of immunization in the elderly [21]. Circadian rhythms act to optimize physiological activities and metabolic health via temporal coordination of cell function, tissue function, and behavior. However, these internal rhythms weaken with age, thus affecting the body’s metabolism and immunity [22,23]. Nonetheless, the impact of age-related long-term circadian rhythm disturbances on spleen immune function are unclear. Hence, the current study demonstrated the effects of double-knockout Per1/Per2 on the spleen’s immune functions in mice aged 14 months. We observed that 4- and 9-month-old DKO mice had fairly normal spleen structures, and that thinning of the spleen WP and blurring of the marginal area occurred only in 14-month-old DKO mice, suggesting that the Per1/Per2 knockout effects on the spleen tissue merged with age.

The interactions between T and B lymphocytes are central to the regulation of immune functions [24]. Flow cytometry and immune cell infiltration analyses showed that the numbers of T and B lymphocytes in the spleen of 14-month-old DKO mice were significantly lower than those in the spleen of WT mice of the same age. This indicates that long-term circadian disturbances could affect lymphocyte numbers and immune functions in mice. We also found that the numbers of mast cells were significantly higher in the spleens of DKO mice than of those in the spleens of WT mice [25]. Mast cells are driven by circadian clocks and allergic responses [26,27]. The abnormal production of mast cells results in the development of allergies [28]. Thus, this result suggests that long-term circadian abnormalities could predispose individuals to develop allergies, not only by activating mast cells in the spleen, but also but also by causing mastocytosis, both of which cause oxidative stress and heightened inflammatory responses. However, the detailed mechanisms of these processes need to be further explored.

We then analyzed DEmRNAs based on transcriptome sequencing data, followed by pathway analysis. GO analysis indicated that most of the DEGs identified were involved in DNA damage repair-related pathways, including DNA repair (GO-BP), ATPase activity (GO-MF), and chromosomal region (GO-CC). KEGG and GSEA analyses indicated that genes related to immuno-regulation and oxidative stress resistance were also enriched. Research indicated that in radiation therapy for tumors, DNA damage is rapidly detected by the ATM (ataxia telangiectasia-mutated gene) and ATR (ataxia telangiectasia and Rad3-related) serine/threonine kinases. These kinases initiate the DDR (DNA damage response) signaling cascades that activate the downstream checkpoint kinases 1/2 (CHEK1/2), which then arrest the cell cycle so that the DNA can be repaired [29]. Oxidative DNA damage can also lead to ferroptosis, caused by CD36-mediated PUFA uptake [30,31], acetyl-CoA carboxylase (ACAC)-dependent PUFA synthesis [32], or lipophagy-induced PUFA production [33]. At the molecular level, multiple regulators may be involved in ATM- or p53-mediated crosstalk between ferroptosis and other regulated cell death (RCD) responses that are activated when DSBs occur [14].

Transcriptomic analysis indicated that the expression levels of ATM, ATR, CHEK1/2, CD36, RB, ACSL4, and LPCAT, which are involved in detecting/repairing DNA damage and mediating ferroptosis [14], were significantly up-regulated in the DKO mice (Table S1). It is worth noting that we obtained similar expression profiles comparing the spleen tissues of DKO mice to tissue exposed to radiation therapy used to treat tumors. Our results indicate that long-term disturbances in circadian rhythms damage the spleen in a similar way to how radiotherapy damages tumor cells—by activating ferroptosis via oxidative damage and decreased immune function.

We next explored the molecular mechanism that connect the loss of Per1/Per2 gene expression with the occurrence of ferroptosis in the spleen cells of aging mice. It is known that ROS and MDA production promote cell death via ferroptosis [34,35], and that both are signals produced by aging bodies [36] in response to abnormal circadian rhythms [37]. In our study, spleen cell mortality and levels of ROS, and MDA were all significantly higher in DKO spleen lymphocyte than in WT spleen lymphocyte. In addition, the spleen lymphocytes from DKO mice were more sensitive to erastin than those of WT mice, which indicates that the loss of Per1/Per2 reduces spleen lymphocyte tolerance to oxidative damage. Correspondingly, the up-regulation of Tfr1, Prnp, Steap3, and Slc39a8 in 14-month-old DKO mice showed that iron metabolism and iron transport were enhanced in the spleens of the DKO mice. In addition, the down-regulation of Ftl1 and Tulp4 in these mice indicated abnormal iron storage, which could increase the production of ROS in the spleen cells due to the Fenton reaction [19,38].

Research by Tang et al. [39] indicated that SQSTM1 is responsible for autophagic degradation of ARNTL/BMAL1. The degradation of ARNTL can promote EGLN2 (Egl-9 family hypoxia inducible factor 2) transcription, which leads to the proteasomal degradation of HIF1A. The decrease in HIF1A levels limits lipid storage and promotes lipid peroxidation and ferroptosis. In our study, the expression levels of ARNTL/BMAL1, SQSTM1, and EGLN2 were significantly lower in 14-month-old DKO mice than those in WT mice; however, the expression level of HIF1A remained unchanged (Figure S5A,B and Table S1). We found that the Ube2o (ubiquitin-conjugating enzyme E2 O) [40] and Ube3a (ubiquitin protein ligase E3A) [41] genes (both of which inhibit ARNTL levels) and Sumo1 and Sumo2 [42] genes (both of which protect the stability of HIF-1α protein) were up-regulated in the spleens of DKO mice (Figure S5B). Thus, we predict that ferroptosis in the spleens of DKO mice may not be triggered by the clock gene, and that other molecular mechanisms may be responsible for ferroptosis.

The accumulation of iron-dependent lipid peroxidase [43,44], which can detoxify lipid peroxides and maintain them at non-toxic levels, is an important defense against ferroptosis. There is a constant battle between anti-ferroptosis systems (including both GPX4-dependent and -independent systems) and pro-ferroptosis systems (including PUFA-PL metabolism and peroxidation, and iron metabolism). At the transcriptome level, we found that pro-ferroptosis genes such as Acsl4 and Lpcat3 were significantly up-regulated in the DKO mice, whereas the expression levels of GPX4 were unchanged. To confirm whether Per1/Per2 DKO affects spleen ferroptosis defense systems, we investigated the expression levels of GPX4 protein. Our results indicated that the expression levels of GPX4 proteins, upstream of the RORγ-SREBP2 pathway, and those of the mevalonate pathway were significantly lower in DKO mice than in WT mice. The levels of key enzymes such as HMGCR, MVD, TRIT1, and SBP2 were also lower in DKO mice than in WT mice. This indicates that Per1/Per2 DKO-induced circadian disruption can inhibit the translation of GPX4; this affects the ferroptosis defense systems in the spleen and predisposes the spleen cells to death via ferroptosis.

Studies have revealed that ferroptosis plays a direct role in the immune response, and that immune cells undergo ferroptosis under specific conditions, causing impaired immune activity [45]. These facts, combined with our work, lead us to believe that immune cell ferroptosis increased in Per1/Per2 DKO mice in old age (14 months), and is responsible for the decreases in immune cell numbers, affecting the spleen’s immune function in mice. However, the C57BL/6J strain of mice used in our work is deficient in the biosynthesis of melatonin, an important effector of circadian clocks in the brain and retina, which might have affected our results [46]. Hence, in future studies, our experiments need to be replicated in other mice strains to further elucidate the impacts of circadian rhythm disturbances on the spleen’s immune functions.

## 4. Materials and Methods

### 4.1. Animals

C57BL/6J mice with knockout of the *Per1* and *Per2* genes (DKO, *n* = 20) were bred from *Per1+/−/Per2+/−* heterozygotes (purchased from the Experimental Animal Center of Soochow University), as described previously [47]. Mice aged 4, 9, and 14 months were housed in a clean laboratory environment at a room temperature of 22 ± 2 °C, relative humidity of 60 ± 10%, and a 12 h light/12 dark cycle at a density of 2–3 mice per cage. Mice had access to laboratory chow and water ad libitum. This experiment was carried out according to the Guidelines for the Care and Use of Experimental Animals and approved by the Experimental Animal Ethics Committee of Wuhan Light Industry University (ID: 20210705006). At the end of the experiment, mice were fasted for 12 h and sacrificed by CO_2_ asphyxiation. Blood was collected in heparin sodium EP tubes (1.5 mL) and stored at 4 °C. Spleens were harvested, weighed, and cut into two portions, with one portion stored at −80 °C for transcriptome analysis and the other fixed with 4% paraformaldehyde and embedded in paraffin for histological studies.

### 4.2. Histological Study and Immunohistochemical Staining

#### 4.2.1. Histology

Paraformaldehyde-fixed, paraffin-embedded tissue was dehydrated with an ethanol gradient and sliced into thin sections by microtome for hematoxylin–eosin (HE) staining. Histopathological changes were observed by the OLYMPUS-BX51 microscope.

#### 4.2.2. Immunohistochemistry

Sections were dewaxed, incubated with 3% H_2_O_2_ at room temperature to eliminate endogenous peroxidase activity, washed with distilled water, soaked in 1× phosphate-buffered saline (PBS), and sealed with 5–10% normal goat serum before incubation with anti-TFR1 antibody (1: 1000, 10084-2-AP, Proteintech, Rosemont, IL, USA) overnight at 4 °C. Following a wash with PBS, HRP-labeled secondary antibody was added with incubation at 37 °C. The tissue was washed with PBS and re-stained with DAB chromogenic agent. Immunohistochemical changes were observed by the OLYMPUS-BX51 microscope.

### 4.3. Flow Cytometry

All spleens were removed under sterile conditions; the spleen capsule was excised and the tissue was cut into small pieces with ophthalmic scissors. Spleen tissue was ground with a syringe piston on a cell filter screen after the addition of buffer. After grinding, the screen was washed with tissue diluent to collect the cell suspension and filter. Spleen lymphocytes were prepared from a 10^8^~10^9^ cells/mL suspension using a mouse spleen lymphocyte isolation kit, according to the manufacturer’s instructions. Blood was diluted with buffer (1:1) and mononuclear cells were isolated using a mouse blood lymphocyte isolation kit, according to the manufacturer’s instructions. Cell suspensions (10^8^~10^9^ cells/mL) were incubated with FITC anti-mouse CD3+ monoclonal, PE anti-mouse CD4+ monoclonal, APC anti-mouse CD8+ monoclonal, and APC anti-mouse CD19 monoclonal antibodies (all from Biolegend, USA) according to the manufacturer’s instructions and detected by a BECKMAN COULTER CytoFLEX flow cytometer. All data were analyzed by Kaluza.2.1 software (version: 2.1) (Becton Dickinson Beckman Coulter, Brea, CA, USA), and at least 10,000 events/samples were obtained.

### 4.4. BGISEQ-500 RNA-Seq

Spleen transcriptome sequencing of 14-month-old WT and DKO mice was commissioned to BGI Medical Laboratory Co., Ltd. Total RNA was extracted from the spleen samples of each mouse and three samples from each group were pooled (100 mg/sample). RNA integrity was confirmed using an Agilent 2100 bioanalyzer before cDNA library construction.

Target sequences were purified and enriched by PCR amplification and samples from Qubit quantitative PCR were pooled to make a single-stranded DNA loop (ssDNA loop) for library construction. Replication of ssDNA was used to generate DNA nanospheres and amplify the fluorescence signal during sequencing. DNA was loaded into a graphical nanoarray, and a 50 bp single-ended read was taken on the BGISEG-500 platform for subsequent data analysis studies.

Reference genome data were derived from the NCBI database (Mus musculus version GCF_000001635.26_GRCm38.p6 https://www.ncbi.nlm.nih.gov, accessed on 29 December 2020). The differential gene expression was analyzed by edgeR (version 3.34.1). Differentially expressed genes (DEGs) met the following criteria: log2 (fold change) > 0.1; *p*-value < 0.05. GO, KEGG, and GSEA pathway enrichment analyses were performed. The R package “mMCP-counter” (version 1.1.0) was used to quantify immune and stromal cell populations.

### 4.5. Cell Culture

A 10^8^–10^9^ cells/mL suspension of spleen lymphocytes was prepared from 14-month-old WT and DKO mice, cultured in RPMI 1640 medium containing serum (10,491, Solarbio, Beijing, China). Lymphocytes were incubated with PBS (pH 7.4) for 12 h as the control group, and 0.5 µM erastin (HY-15763, MCE, Princeton, NJ, USA) for 12 h as the experimental group according to the manufacturer’s instructions. Cell mortality was measured with Trypan blue solution (72-57-1, Solarbio, Beijing, China).

### 4.6. MDA and ROS Assays

#### 4.6.1. MDA

Following incubation with erastin and PBS, lymphocytes were sonicated to disrupt the cell integrity and MDA was measured using a malondialdehyde (MDA) detection kit (BC0025, Solarbio, Beijing, China), according to the manufacturer’s instructions. The assay relies on the reaction between MDA and thiobarbituric acid (TBA) to generate an MDA–TBA adduct. Absorbances were simultaneously measured at 600 nm, 532 nm, and 450 nm, and MDA content was calculated from the absorbance differentials.

#### 4.6.2. ROS

Following incubation with erastin and PBS, 10^6^ cells/mL lymphocytes were suspended in diluted DCFH-DA and incubated at 37 °C for 20 min before washing three times with serum-free cell culture solution. An ROS detection kit was used (CA1410, Solarbio, Beijing, China), according to the manufacturer’s instructions, with flow cytometry to detect and calculate ROS levels.

### 4.7. Western Blot Analysis

Total protein was extracted and quantified using a BCA protein assay kit (A8020, Solarbio, Beijing, China) and mixed with sample buffer in a ratio of 1:1 (*v*/*v*). Samples were incubated in boiling water for 5 min, and 30 mg of protein from each sample were separated by 12.5% SDS-PAGE gel electrophoresis at a constant voltage (120 V) and transferred to a PVDF membrane. The membrane was sealed with 5% skim milk and incubated overnight at 4 °C with the primary antibodies shown in Table S2. β-actin was used as a loading control. Optical densities were analyzed quantitatively by Image J software.

### 4.8. qPCR Analysis

Lymphocyte total RNA was extracted using Trizol reagent and concentrations were assessed by measurements of absorbance at 260/280 nm. cDNA was synthesized from 5 µg total RNA using SuperScript IV reverse transcriptase, and quantitative PCR was performed in a total volume of 20 µL with 300 nM reverse and forward primers (Table S3) using a CFX96 real-time PCR system (Bio-RAD, Hercules, CA, USA). Thermal cycling conditions were as follows: 2 min at 50 °C, 3 min at 95 °C, 40 times at 95 °C for 15 s, 60 times at 30 °C, 72 times at 30 °C. Target mRNA was normalized to the expression of β-actin, and fold changes were expressed by 2^−ΔΔCt^ values. The purity of the PCR product was verified by the melting curve.

### 4.9. Statistical Analysis

All data are presented as means ± SD with normal distribution. The significant differences between DKO and WT mice in transcriptome expression levels of all genes were annotated according to the results of differential gene analysis using the R package “edgeR” (version 3. 34.1), shown in Figure 6B, Figure 7C and Figure S4 (RNA-seq), and Figure S5B; the predicted *cor_pvalue* for immune cells is based on the calculation of the R package “mMCP-counter” (version 1.1.0). The significant differences among four groups were determined by one-way ANOVA using GraphPad Prism (version 7.0), shown in Figure 6 C–E. The significant differences between two groups were determined by the multiple *t* test-one per row using GraphPad Prism (version 7.0), shown in Figure 1A,B, Figure 2, Figure 5A, Figure 6F, Figure 7B,D and Figure S4 (RT-qPCR), and Figure S5B. Differences were considered statistically significant if *p* < 0.05.

## 5. Conclusions

In summary, knockout of the circadian rhythm genes, *Per1* and *Per2*, induced the expression of DNA damage repair genes and increased the incidence of ferroptosis in spleen lymphocytes. These processes changed the expression profiles of several genes in the following manner: (1) increased expression of *Cd36*, *Acsl4, Acsl1*, and *Lpcat3*, which up-regulated PUFA-PL and PUFA-ePLs synthesis; (2) increased expression of TFR1 and other iron transport proteins, which led to an increase in the levels of ferritin-bound iron into the labile iron pool, thereby sensitizing cells to ferroptosis; (3) increased the expression of *Rb*, *Atm*, *Atr*, and *Ampk*, which up-regulated *SLC7A11* and *SLC3A2*, and suppressed GPX4; (4) decreased *Rorγ* activity, which inhibited the expression of Srebp2, and decreased the expression levels of HMGCR, MVD, TRIT1, and SBP2 proteins, which in turn suppressed GPX4 RNA translation.

## Data Availability

The data presented in this study are available on request from the corresponding author.

## Supplementary Materials

The following supporting information can be downloaded at: https://www.mdpi.com/article/10.3390/ijms232112962/s1.
