# Supplementary material for: Per1/Per2 knockout Affects Spleen Immune Function in Elderly Mice via Inducing Spleen Lymphocyte Ferroptosis
Source: Int J Mol Sci. 2022 Oct 26;23(21):12962. doi: 10.3390/ijms232112962 (PMC9657961; doi:10.3390/ijms232112962)
Supplement: Supplementary file 1 [file ijms-23-12962-s001.zip › ijms-1905667-supplementary.pdf]

Supplementary Data

Per1/*Per2* Knockout Affects Spleen Immune Function in Elderly Mice via Inducing Spleen Lymphocyte Ferroptosis

Ruyi He †, Shijie Zhang †, Jiale Yu, Xiaojie Yu, Jian Wang, Yi Qiu, Wenting Zhou, Fangyi Wang, Feng Ren and Zhiguo Liu *

School of Life Science and Technology, Wuhan Polytechnic University, Wuhan 430024, China
* Correspondence: zhiguoliu@whpu.edu.cn
† These authors contributed equally to this work.

This PDF file includes:

Supplementary Tables S1–S3

Supplementary Figures S1–S5

Table S1. Changes in the ratio of DKO/ WT-related differential mRNAs in ferroptosis pathways

Gene Symbol	Description	logFC	P.Value	change
Atr	Ataxia telangiectasia and Rad3 related	0.772927	2.94E-06	UP
Atm	Ataxia telangiectasia-mutated gene	0.392001	0.006306	UP
Prkdc	Protein Kinase, DNA-Activated, Catalytic Subunit	0.473959	0.000732	UP
Chek1	Checkpoint Kinase 1	1.801378	4.35E-16	UP
Chek2	Checkpoint Kinase 2	0.976704	1.37E-09	UP
Apex2	Apurinic/Apyrimidinic Endodeoxyribonuclease 2	0.333752	0.030491	UP
Msh6	MutS Homolog 6	0.635101	3.82E-06	UP
Pms2	PMS1 Homolog 2, Mismatch Repair System Component	0.440204	0.00027	UP
Dnmt1	DNA Methyltransferase 1	0.432902	0.015051	UP
H2afx	H2A histone family, member X	0.805355	0.000103	UP
Msh5	MutS Homolog 5	0.417796	0.030714	UP
Msh3	MutS Homolog 3	0.842693	9.58E-10	UP
Bcl6	BCL6 Transcription Repressor	0.036382	0.794541	NOT
Gadd45b	Growth Arrest And DNA Damage Inducible Beta	-0.37955	0.008765	DOWN
Sod3	Superoxide Dismutase 3	-0.73053	8.73E-05	DOWN
Smad4	SMAD Family Member 4	-0.2693	0.034612	DOWN
Smad3	SMAD Family Member 3	-0.28746	0.019942	DOWN
Foxo1	Forkhead Box O1	-0.4173	0.012844	DOWN
Klf2	Kruppel Like Factor 2	-0.4301	0.013976	DOWN
Sirt1	Sirtuin 1	0.89069	7.00E-08	UP
Foxo4	Forkhead Box O4	0.337196	0.007948	UP
Foxo3	Forkhead Box O3	0.788951	0.000103	UP
Cd36	CD36 Molecule	0.844657	2.27E-06	UP
Acsl4	Acyl-CoA Synthetase Long Chain Family Member 4	0.500407	0.002451	UP
Lox	Lysyl Oxidase	0.569453	0.039129	UP
Lpcat3	Lysophosphatidylcholine Acyltransferase 3	0.887262	3.15E-07	UP
Steap3	STEAP3 Metalloreductase	1.077301	2.53E-06	UP
Slc11a2	Solute Carrier Family 11 Member 2	0.763997	2.73E-06	UP
Ireb2	Iron Responsive Element Binding Protein 2	0.741732	4.40E-06	UP
Tulp4	TUB Like Protein 4	-0.25114	0.032295	DOWN
Slc39a8	Solute Carrier Family 39 Member 8	1.586668	2.93E-09	UP
Tfrc	Transferrin Receptor	2.454485	9.12E-10	UP
Prnp	Prion Protein	0.472854	0.005242	UP
Ftl1	Fms related tyrosine kinase 1	-0.2649	0.036538	DOWN
Rorc	RAR Related Orphan Receptor C	-0.92871	8.70E-05	DOWN

Srebf2	Sterol Regulatory Element Binding Transcription Factor 2	-0.43092	0.015567	DOWN
Gpx4	Glutathione Peroxidase 4	0.132068	0.487136	NOT
Sepsecs	Sep (O-Phosphoserine) TRNA:Sec (Selenocysteine) TRNA Synthase	0.407574	0.044122	UP
Secisbp2	SECIS Binding Protein 2	0.042339	0.730102	NOT
Trit1	TRNA Isopentenyltransferase 1	-0.17204	0.169288	NOT
Hmgcr	3-Hydroxy-3-Methylglutaryl-CoA Reductase	0.149581	0.212724	NOT
Mvd	Mevalonate Diphosphate Decarboxylase	-0.31105	0.051668	NOT
Sumo1	Small ubiquitin like modifier 1	0.451489	0.0005123	UP
Sumo2	Small ubiquitin like modifier 2	0.478137	0.0070311	UP
Ube2o	ubiquitin conjugating enzyme E2 O	0.698306	0.0150987	UP
Ube3a	Ubiquitin Protein Ligase E3A	0.671174	1.66E-05	UP
Arntl	Aryl Hydrocarbon Receptor Nuclear Translocator Like	-0.51523	0.000585	DOWN
Sqstm1	Sequestosome 1	-0.34414	0.049441	DOWN
Egln2	Egl-9 Family Hypoxia Inducible Factor 2	-0.32685	0.005514	DOWN
Hif1a	Hypoxia Inducible Factor 1 Subunit Alpha	0.414608	0.002703	UP

Table S2. Antibodies used in this study

Protein	Manufacturer	LOT.NO
GPX4	proteintech	67763-1-Ig
SLA	Proteintech	11551-1-AP
SBP2	Proteintech	12798-1-AP
IPT	BIOSS	bs-17134R
HMGCR	abcam	ab174830
MVD	Proteintech	15331-1-AP
BMAL1	Santa	SC-373955
SQSTMI	Proteintech	18420-1-AP
EGLN2	Affmity	DF7918
HIF1A	Santa	SC-13515
β-actin	Rabbit	abcam

Table S3. qPCR primers used in this study

Gene	Forward Primer（5- 3）	Reverse Primer（3-5）
β-actin	CGTTGACATCCGTAAAGACC	AACAGTCCGCCTAGAAGCAC
Rorγ	TGCGACTGGAGGACCTTCTACG	GTTGTTGGACGGGCTGAGGATG
Srebp2	CACAAGTCTGGCGTTCTGAGGAAG	CCAGCTTCAGCACCATGTTCTCC

SUPPLEMENTARY FIGURES

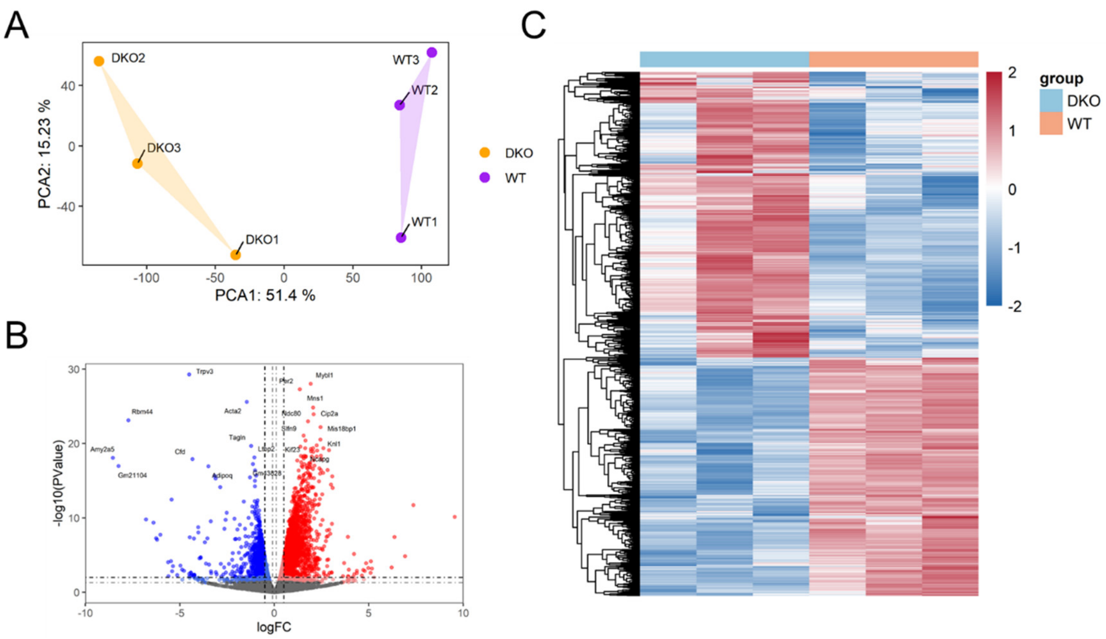

Figure S1. Differentially expressed spleen RNA between 14-month-old DKO and WT mice (|log2 fold change| >0.1, *p.value* < 0.05). Compared with normal samples, 3583 mRNA were upregulated and 2290 mRNA were downregulated in DKO mice. (**A**) Principal component analysis (PCA) of DKO samples and WT samples; (**B**) Volcano map for differentially expressed mRNAs. Red stands for up-regulation, blue stands for down-regulations, and gray stands for intermediate in volcanoes. Each point represents a gene. (**C**) Heatmap for differentially expressed mRNAs. Red stands for DKO male mice, and blue stands for WT male mice.

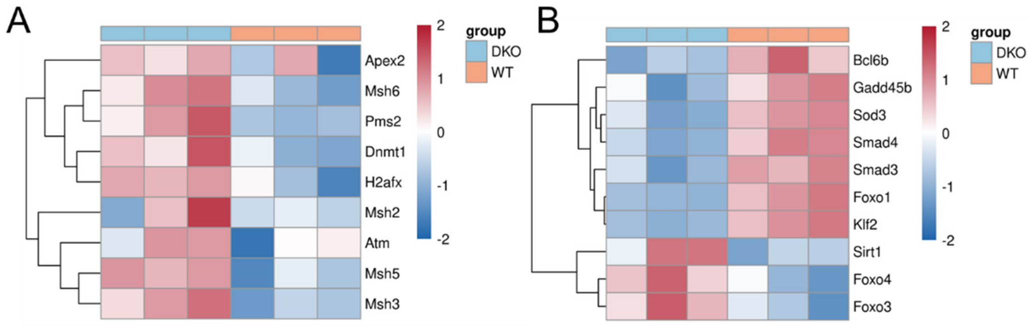

Figure S2. Heatmap for DEmRNAs involved in (**A**) DNA repair pathways, (**B**) FoxO signaling pathway. Blue: DKO mice; Red: WT mice.

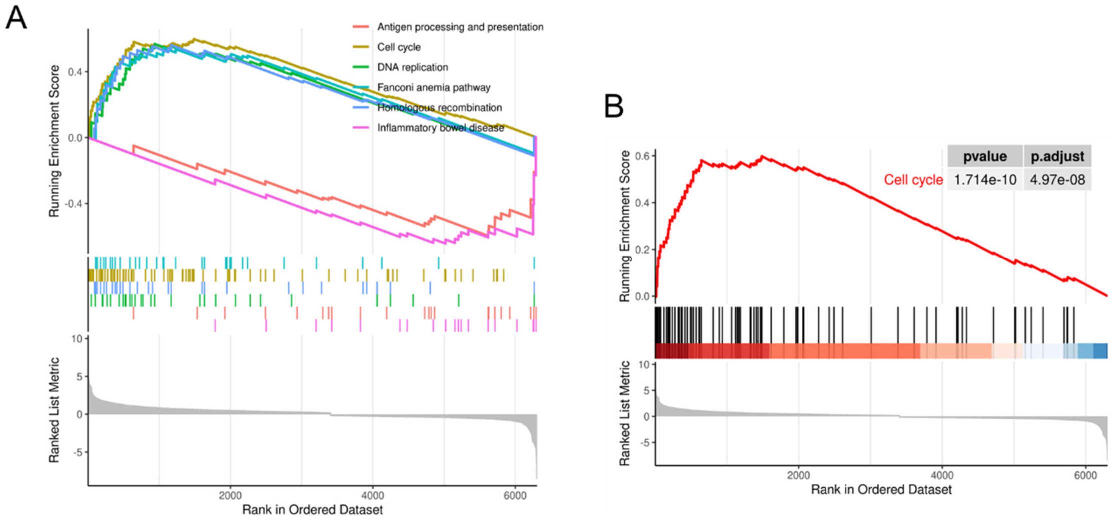

Figure S3. Gene set enrichment analysis (GSEA) for DEmRNAs

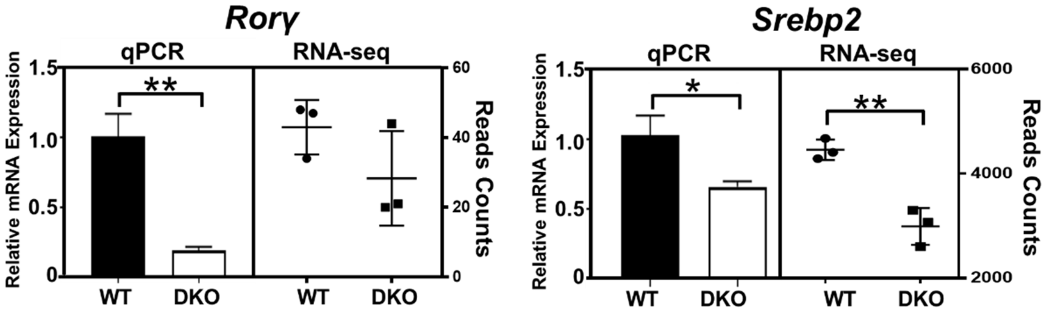

Figure S4. RT-qPCR results (left side of each image) and mRNA relative expression in Reads counts (right side of each image) of *Rorγ* and *Srebp2*. Data are presented as mean ± SD, n = 3/group. * $p < 0.05$, ** $p < 0.01$.

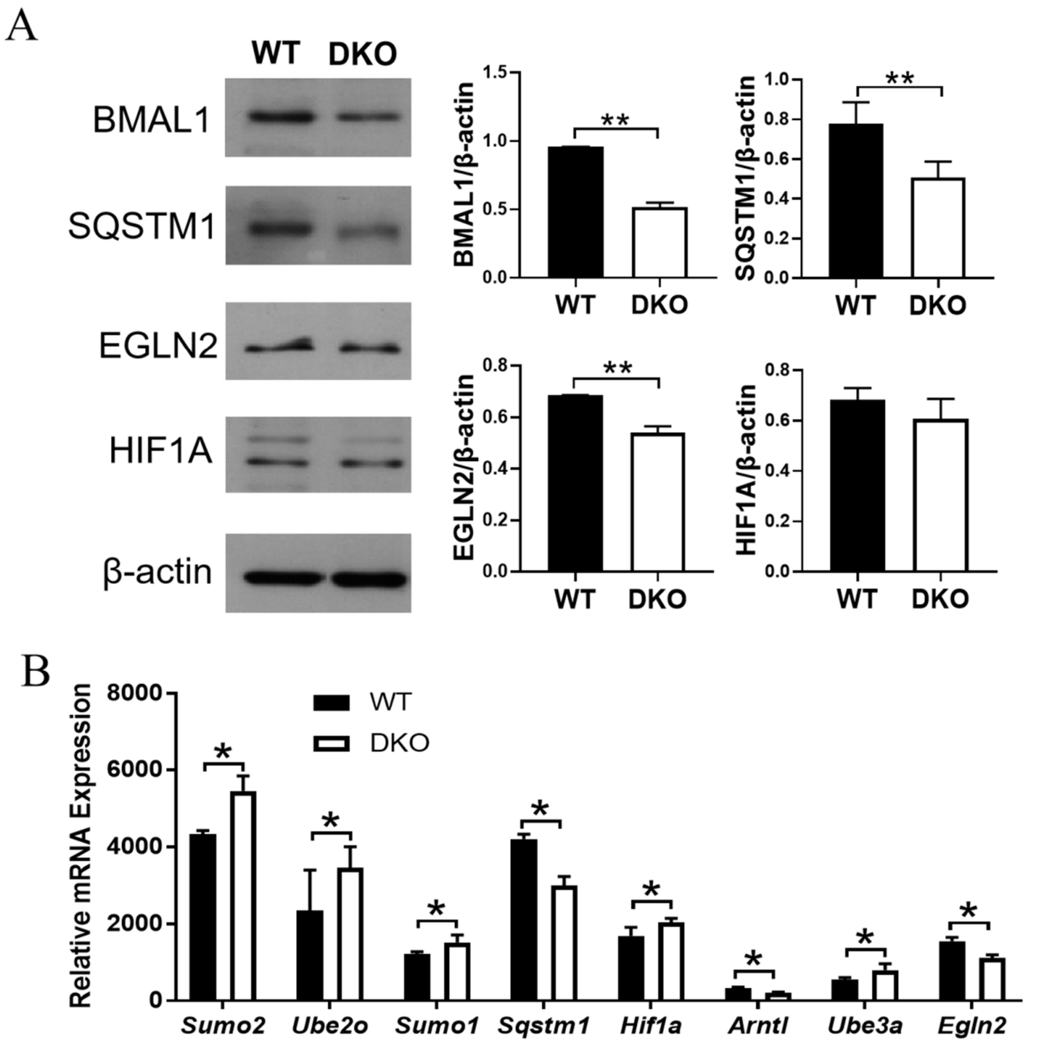

Figure S5. Expression of selective autophagic degradation related genes. (**A**) Western blot results of Bmal1, SQSTM1, EGLN2, and HIF1A. (**B**) Relative mRNA expression of *Sumo2*, *Ube2o, Sumo1, Sqstm1, Hif1a, Arntl, Ube3a, Egln2*. Data are presented as mean ± SD, n = 2/group.* $p < 0.05$, ** $p < 0.01$.
